# Effectiveness of online advanced C.A.R.E suicide prevention gatekeeper training program among healthcare lecturers and workers in national university of Malaysia: A pilot study

**DOI:** 10.3389/fpsyt.2023.1009754

**Published:** 2023-01-19

**Authors:** Amran Fadzrul Roslan, Kai Shuen Pheh, Raynuha Mahadevan, Siti Mariam Bujang, Ponnusamy Subramaniam, Hanieza Fadzlina Yahya, Lai Fong Chan

**Affiliations:** ^1^Department of Psychiatry, Faculty of Medicine, National University of Malaysia (UKM), Kuala Lumpur, Malaysia; ^2^Department of Psychology and Counselling, Faculty of Arts and Social Sciences, Tunku Abdul Rahman University, Kampar, Malaysia; ^3^Department of Psychiatry, Hospital Canselor Tuanku Muhriz, National University of Malaysia (UKM), Kuala Lumpur, Malaysia; ^4^Department of Medical Education, Faculty of Medicine, National University of Malaysia (UKM), Kuala Lumpur, Malaysia; ^5^Unit of Health Psychology, Faculty of Health Sciences, National University of Malaysia (UKM), Kuala Lumpur, Malaysia

**Keywords:** suicide prevention, gatekeeper training, effectiveness study, online intervention, healthcare

## Abstract

**Background:**

Suicide is a major cause of death among adolescents and young adults, especially students. This is particularly true for healthcare students with a higher risk and more access to lethal means. Thus, it is vital for healthcare educators who have regular contact with these healthcare students to be trained as gatekeepers in preventing suicide. Evidence of the effectiveness of such gatekeeper training, mainly using an online module, is lacking predominantly in Malaysia. This study aims to investigate the effectiveness of an online gatekeeper suicide prevention training program that is conducted for healthcare lecturers.

**Methods:**

A single-arm interventional pre-and post-pilot study was conducted on a sample of healthcare lecturers and workers who are involved in supervising healthcare students. A purposive sampling technique was used to recruit 50 healthcare educators in Malaysia. The program was conducted by trained facilitators and 31 participants completed a locally validated self-rated questionnaire to measure their self-efficacy and declarative knowledge in preventing suicide; immediately before and after the intervention.

**Results:**

Significant improvement was seen in the overall outcome following the intervention, mostly in the self-efficacy domain. No significant improvement was seen in the domain of declarative knowledge possibly due to ceiling effects; an already high baseline knowledge about suicide among healthcare workers. This is an exception in a single item that assesses a common misperception in assessing suicide risk where significant improvement was seen following the program.

**Conclusion:**

The online Advanced C.A.R.E. Suicide Prevention Gatekeeper Training Program is promising in the short-term overall improvement in suicide prevention, primarily in self-efficacy.

## Introduction

There were 703,000 suicide cases each year and it has been the fourth leading cause of death for 15–29 years old globally (1) and the Malaysian National Suicide Registry (NSRM) dated from 2007 to 2009 has reported that the highest suicide rate is within this age group (2–4). A more recent study has shown a suicide prevalence of 6–8 per 100,000 population per year in Malaysia (5). Being school leavers put them at high risk of suicide (6–10) and it has also been reported that they are the group with the highest risk to have mental health problems (11). Furthermore, these students, especially Malaysian healthcare students (12) are less likely to seek professional help when depressed (13–15) or having suicidal thoughts.

In Malaysia, it is estimated that 5 deaths by suicide occur every day (16). From a global and cultural lens, studies have shown that religion can be a protective factor against suicide, especially among Muslims (17, 18). It is interesting to note that the average suicide rate in Malaysia is the second highest in comparison to other countries with predominantly Muslim populations in the Middle East and Indonesia (16). According to Lew et al. the heterogeneity of Malaysia’s religion and ethnicity might influence the suicide rate whereby Malaysia has the lowest percentage of Muslims (61.3%) compared to other Muslim-majority countries (16). In addition, Malaysian students population are an at-risk population for suicidal behavior (10). More studies on the suicide rate in other countries have found a higher suicide rate among healthcare workers and healthcare students compared to other professions (12, 19–22) due to multiple factors. This has also been reflected in a Malaysian study. It was estimated that 11% of healthcare workers including healthcare students are reported to have suicidal ideation, particularly those in the early phase of their careers (23). As suicide is preventable, multiple suicide prevention measures have been developed including gatekeeper training. It aims to increase the chances of individuals at risk of suicide being approached, connected with, and referred for help and support (15, 24, 25). It has been found that almost half of suicide victims have communicated their intentions before the act (26, 27) but failure in judging their intentions at that time will lead to misunderstanding and closure of communication (28) that will eventually lead to suicide. Gatekeepers in suicide prevention refer to “individuals in a community who have face-to-face contact with large numbers of community members as part of their usual routine” (29). Having gatekeepers at education centers also promotes hope and wellbeing among college students (30, 31), signaling to them that help is within their reach. Without proper training, it can be difficult to detect someone with active suicidal thoughts as the thoughts may be present even without apparent symptoms (32, 33).

As part of suicide prevention measures, the WHO has long been recommending that school staff should undergo training (34) to qualify them to be a gatekeeper. This task is usually appointed to the teachers (35), who are the closest to those students during the school period. Appointable teaching staff gatekeepers include university lecturers or academic supervisors especially those in healthcare education (25, 33). Many forms of gatekeeper training may increase knowledge and self-efficacy on suicide prevention (36), enhancing trainee gatekeepers’ confidence in talking about suicide (7, 15, 37). A study shows that this training outcome may be effective for at least a month (38) or even longer in self-efficacy in preventing suicide (39).

C.A.R.E Suicide Prevention Gatekeeper Training Program (40, 41) is a program designed to train individuals who are potentially exposed to those with suicidal thoughts. It has four core principles in handling cases related to suicidal thoughts. It can be easily memorized with the acronym CARE which stands for (i) Catch the signs; (ii) Acknowledging emotional pain; (iii) Risk formulation; and (iv) Encourage collaborative care. The program has shown its effectiveness in enhancing the awareness of warning signs and building up the confidence of gatekeepers in engaging and handling individuals with suicidal crises (40, 41). The program was then modified, improved, and introduced as Advanced C.A.R.E Suicide Prevention Gatekeeper Training Program (AdCARE). It implements Safety Planning Intervention (42), Ask Suicide-Screening Questions (ASQ) (43), and suicide postvention (44). These programs are novel and valuable tools for gatekeepers in preventing suicide (45, 46). Due to the recent pandemic situation of COVID-19, the program was converted into a 3-h online module to ensure safety for both the participant and the research team. This shift leads to logistic advantages in improving accessibility and better cost-effectiveness.

Our study aims to assess the effects of the online module of AdCARE (Online AdCARE) on healthcare lecturers and workers from various healthcare fields who supervise healthcare students. We hypothesize that the Online AdCARE gatekeeper training program would significantly improve the study participants’ knowledge, attitude, and practice in terms of suicide prevention literacy.

## Materials and methods

### Study design

This was a single-arm pre-and post-test interventional study.

### Study site

In this study, we defined healthcare personnel as those who provide services to patients either directly or indirectly (47, 48). Healthcare personnel comprises various departments within the National University of Malaysia (UKM). At UKM, healthcare students were supervised by the lecturers. Some of the students especially those doing practical duty were being supervised by non-lecturers such as clinicians. Thus, we selected the participants among the lecturers from healthcare faculties in UKM including the Faculty of Medicine, Faculty of Dentistry, Faculty of Pharmacy, and Faculty of Health Sciences. We also included healthcare workers from Hospital Canselor Tuanku Muhriz, Kuala Lumpur, UKM who were involved in supervising healthcare students.

### Sampling and recruitment

Program details were broadcasted through networks of the research team and UKM lecturers *via* emails, instant messaging applications (e.g., WhatsApp and Telegram), social media channels (e.g., Facebook and Twitter), telephone calls, and face-to-face meetings. Digital posters and digital announcements through the UKM system were also utilized to improve recruitment. Digitalized forms were used for recruitment and questionnaires in light of the current pandemic situation.

Inclusion criteria for samples were: (i) All healthcare lecturers and workers from the study site with experience in performing supervision of healthcare students, (ii) had no suicidal thoughts or plans within the past 2 weeks, and (iii) no bereavement of suicide in the past 6 months. The latter two criteria were included for safety considerations, as they might be more vulnerable to experiencing emotional difficulties (49–51), especially with the intense exposure to suicide-related content during the program. Samples were screened through a self-report questionnaire during the invitation and they are provided with relevant help-seeking resources.

We excluded those who were involved in the previous Advanced C.A.R.E. Suicide Prevention AdCARE-Q Validation study (52) and those without experience in supervising healthcare students as part of their duty. We also excluded those who did not complete the Online AdCARE program.

Based on G*POWER Program V3.1 calculation, with power at 0.8 and α level at 0.05, and calculated effect size from a previous study (41) at 0.6775, the minimum sample size calculated was 20 and after considering a 20% attrition rate, the total required to sample for this study is 25 participants. A purposive sampling technique was applied to include all lecturers and healthcare workers from the study sites. An information sheet containing the purpose and explanation of the study was given to all participants and before the study entry, participants provided their informed consent.

Demographic information on sex, age, race, department, years of experience in supervising healthcare students, previous exposure to suicide cases, and previous exposure to suicide intervention programs was collected.

### Instruments and program implementation

#### AdCARE-Q

Advanced C.A.R.E. Suicide Prevention Gatekeeper Training Questionnaire (AdCARE-Q) was meant to assess knowledge gains from gatekeeper training that is adapted from Terpstra et al. (37). This is a self-administered, 15-item questionnaire on a five-point Likert scale (see Appendix, Supplementary Digital Content 1). Items from B1 to B4 and D1 until D5 were measured for Self-Efficacy (SE) while items from C1 until C6 were measured for Declarative Knowledge (DK) of suicide prevention. SE was defined as perceived knowledge about suicide prevention and confidence in the ability and willingness to execute suicide prevention measures. Meanwhile, DK stands for tested knowledge of warning signs and risk factors for suicide and appropriate referrals. Higher scores correspond to higher levels of awareness and attitudes toward suicide prevention. AdCARE-Q has recently been validated among medical lecturers and specialists to measure their suicide prevention training gains for gatekeepers (52). There were no significant differences across the professions of specialist doctors and medical lecturers.

#### Online AdCARE

Seven Facilitators including the research team with psychiatric backgrounds, consisting of a psychiatrist, medical officers, clinical psychologists, and a counselor attended a half-day online session of Training of Trainers for the Online AdCARE program a few weeks before the intervention was held. The program was then held for participants who have been separated into two groups according to their preferred date. The research team led the Online AdCARE program. A total of 20-min of role-play session was held in individual break-up rooms consisting of 1 facilitator to 5 participants in each group. AdCARE-Q was distributed to the participant to be answered individually just before (Pre) and right after (Post) the program.

### Statistical analyses

We used IBM SPSS software, version 26.0. Participant demographics were analyzed using the exploratory data analysis by describing frequency (percentage), and mean (standard deviation. For non-normalized data, the median (interquartile range) was used. The effectiveness of the study was analyzed using paired *t*-tests for items in B1–B5 and D1–D5, SE domain, and overall effectiveness. Meanwhile, items in the C1–C6 and DK domains were analyzed using the Wilcoxon Signed-Rank test. Further analysis such as Multiple linear regression was used to check for potential confounders in an overall score change.

## Results

Fifty participants (mean age = 44.1 years; SD = 7.4) registered for online AdCARE; eighteen participants volunteered to join the program on 26th April 2022 (Group A) while other participants volunteered to join on 20th May 2022 (Group B). A total of nineteen participants were excluded from the study. There was no control group in this study and there were no significant differences in the sociodemographic description in those two groups (Table 1).

**TABLE 1:** Sociodemographic description of participants in the study (*N* = 31).

Variable	Group A (*N* = 17)		Group B (*N* = 14)		*P*-value
	*N*	%	*N*	%	
**Gender**					
Male	6	35.3	2	14.3	0.24^ii^
Female	11	64.7	12	85.7	
**Race**					
Malay	13	76.5	12	85.7	1.00^ii^
Chinese	3	17.6	2	14.3	
Indian	1	5.9	0	0	
**Age^a^**					*0.51^ii^
19–49 years old	12	70.6	8	57.1	0.48^ii^
50–65 years old	5	29.4	6	42.9	
**Organization**					
HCTM	8	47.1	5	35.7	0.053^ii^
Faculty of medicine UKM	9	52.9	7	50.0	
Faculty of health sciences UKM	0	0	2	14.3	
**Clinician^b^**					
Yes	7	41.2	5	35.7	1.00^i^
No	10	58.8	9	64.3	
**Lecturer/mentor^c^**					
Yes	16	94.1	10	71.4	0.15^ii^
No	1	5.9	4	28.6	
**Department**					
Anesthesiology	1	5.9	1	7.1	0.91^ii^
Anatomy	1	5.9	1	7.1	
Biochemistry	1	5.9	1	7.1	
Clinical child psychology	0	0	1	7.1	
Emergency	1	5.9	0	0	
Entomology	0	0	1	7.1	
Health education	0	0	1	7.1	
Medical education	1	5.9	0	0	
Medical entomology	1	5.9	0	0	
Microbiology and immunology	1	5.9	0	0	
Neuroscience	1	5.9	0	0	
Nursing	0	0	2	14.3	
Obstetrics and gynecology	1	5.9	0	0	
Otorhinolaryngology	1	5.9	0	0	
Pediatric surgery	1	5.9	0	0	
Pathology	1	5.9	1	7.1	
Pharmacist	0	0	1	7.1	
Pharmacology	2	11.8	3	21.4	
Physiology	1	5.9	0	0	
**Gender**					
Public health	0	0	1	7.1	
Surgery	2	11.8	0	0	
**Experience in mentoring (years)**					*0.48^ii^
10 years or less	11	64.7	8	57.1	0.72^i^
11 years or more	6	35.3	6	42.9	
**Previous experience with student(s) who have had suicidal attempts?**					
Yes	3	17.6	3	21.4	1.00^ii^
No	14	82.4	11	78.6	
**Previous exposure to suicide prevention program**					
Yes	1	5.9	0	0	1.00^ii^
No	16	94.1	14	100	

^a^Classified based on Franssen et al. (75).

^b^Clinician: working directly with patients (participants from anesthesiology, clinical child psychology, emergency, obstetrics and gynecology, pediatric surgery, nurses).

^*c*^Lecturer/mentor: primarily working as lecturer/mentor (specialist doctors-lecturer, lecturers).

*Comparison as continuous variables.

^*i*^Chi-square test.

^*ii*^Fisher’s exact test.

Eight percent (*n* = 4) of the participants were unable to attend Online AdCARE due to unforeseen circumstances. Intriguingly, twenty-two percent (*n* = 11) of participants were excluded as they were not from our study site. Another four percent (*n* = 2) of the participants were also excluded for not having any experience in supervising healthcare students. Forty-two percent (*n* = 13) of the study respondents were supervising healthcare students while primarily working in HCTM and sixty-two percent (*n* = 19) of them were non-clinicians who do not usually work directly with patients and are aware of HCTM standard operating procedures in handling patients with suicidal risk. Participants included in the study have an average of almost 10 years (mean = 9.9 years; SD = 6.5) of supervising healthcare students. Nineteen percent (*n* = 6) of them reported having encountered students with suicidal thoughts, or attempted suicide. However, only 1 of them was exposed to a suicide prevention program.

**FIGURE 1:**
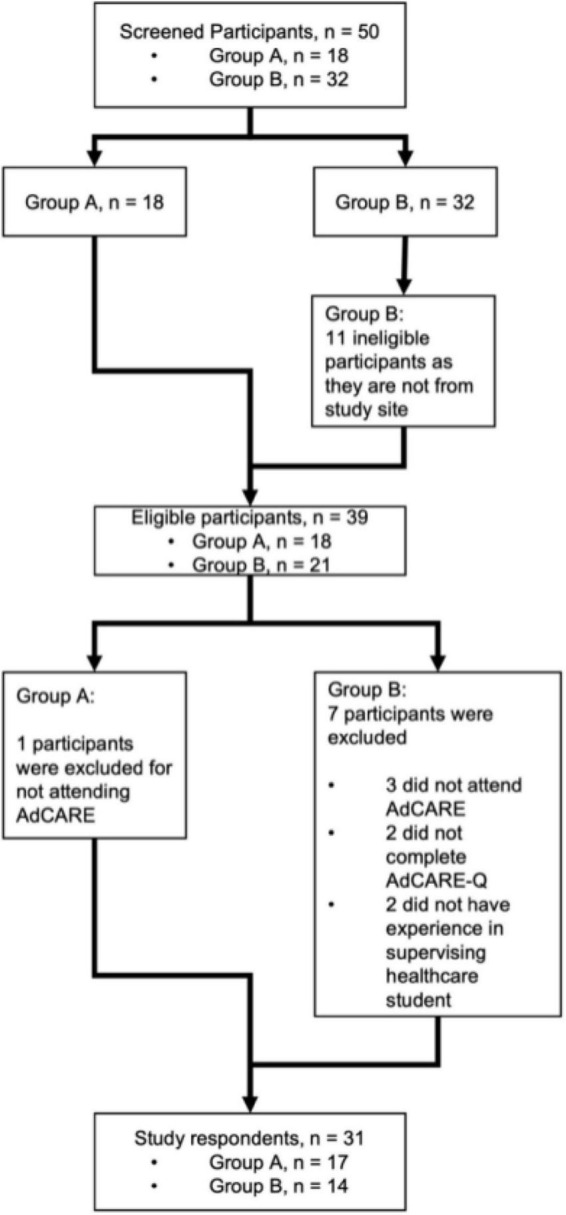
Study flowchart.

Using Chi-Square and Fisher’s Exact tests, it was found that there were no significant differences for all categorical demographic variables between group A and group B. During the program, there were no dropouts. However, four percent (*n* = 2) were excluded from the study as they did not complete AdCARE-Q for an undisclosed reason (Figure 1).

Descriptive analysis of the pre-test AdCARE-Q on the samples showed a high baseline score in most of items of DK; C1 (pre-Mdn = 4.0, post-Mdn = 5.0); C2 (pre-Mdn = 4.0, post-Mdn = 5.0); C3 (pre-Mdn = 5.0, post-Mdn = 5.0); C4 (pre-Mdn = 5.0, post-Mdn = 5.0); C5 (pre-Mdn = 4.0, post-Mdn = 5.0); and thus, the statistical analysis in Declarative Knowledge (DK) domain improvement following online AdCARE was not significant. This is not true for item C6 which asked for participants’ agreement to the statement “People who express their suicidal ideation will not attempt suicide.” It is because there was a significant improvement for this item (*p* < 0.05) following online AdCARE. However, it is quite difficult to obtain the significance as the statistical measurement used for the data that is not normally distributed was in Median (Mdn) and Interquartile Range (IQR); C6 (pre-Mdn = 4.0, IQR = 1; post-Mdn = 4.0, IQR = 1). For all other items, there were significant improvements seen item B1–B4 (*p* < 0.001), D1–D5 (*p* < 0.001), Self-Efficacy (SE) domain (*p* < 0.001), and overall scores (*p* < 0.05) following online AdCARE (Table 2).

**TABLE 2:** Participants’ pre-and post-online AdCARE scores.

No.	Question items	Pre-AdCARE scores		Post-AdCARE scores		Intervention effect		
		Mean (median)	SD (IQR)	Mean (median)	SD (IQR)	T (Z)	*d*f	*P*-value
Self-efficacy (SE)		21.81	6.49	34.74	3.50	14.144	30	** < 0.001
B1	Knowledge on suicide prevention	2.3	0.9	4.0	0.5	11.806	30	** < 0.001
B2	Warning signs of suicide	2.5	0.7	4.0	0.5	12.473	30	** < 0.001
B3	Communicating with someone who is suicidal	2.0	1.0	4.0	1.0	13.096	30	** < 0.001
B4	How to arrange help for a suicidal person	2.1	0.8	3.9	0.5	14.301	30	** < 0.001
D1	I have confidence in my abilities to recognize warning signs of suicide in people	2.6	1.0	3.9	0.6	9.059	30	** < 0.001
D2	I hesitate to ask a person whether they are suicidal	2.7	1.1	3.4	1.1	3.691	30	** < 0.001
D3	I have confidence in my abilities to arrange for help for someone who is suicidal	2.6	1.0	4.0	0.8	6.840	30	** < 0.001
D4	I am confident in discussing about safety planning with someone who is suicidal	2.5	1.1	3.9	0.6	6.477	30	** < 0.001
D5	I know where to seek resources for postvention services	2.3	0.9	4.0	0.5	11.898	30	** < 0.001
Declarative knowledge (DK)		(26.0)	(4)	(27.0)	(5)	(−1.568)	30	0.117
C1	Depression is a potential suicide risk	(4.0)	(1)	(5.0)	(1)	(−0.696)	30	0.486
C2	People who are suicidal may not see a way out of their problems	(4.0)	(1)	(5.0)	(1)	(−0.206)	30	0.837
C3	A person who shows warning signs of suicide should be referred to a healthcare provider	(5.0)	(1)	(5.0)	(1)	(−0.758)	30	0.448
C4	Crisis helplines should be offered to a suicidal person	(5.0)	(1)	(5.0)	(1)	(−0.082)	30	0.934
C5	Farewell messages or asking for forgiveness unexpectedly are warning signs of suicide	(4.0)	(1)	(5.0)	(1)	(−1.345)	30	0.179
C6	People who express their suicidal ideation will not attempt suicide	(4.0)	(1)	(4.0)	(1)	(−2.336)	30	* < 0.05
Total scores (overall)^a^		47.55	7.36	60.71	6.51	8.58	30	** < 0.001

*p < 0.05. **p < 0.001. ^*a*^Total scores (overall): total score change (post–pre).

Other factors that may affect the outcome are such as being in a different group, age, gender, either being a clinician or a lecturer, years of experience as a student supervisor, previous exposure to students with suicidal thoughts or attempts, and previous exposure to suicide prevention programs have been included in the regression analysis result shown that the factors were not significant as confounders to the overall outcome of the study (Table 3).

**TABLE 3:** Multiple linear regressions for potential confounders.

Variables	Unstandardized Coefficients^a^, B	95% CI	*P*-value
Age	0.739	−8.28 to 9.76	0.87
Gender	1.49	−9.90 to 7.01	0.73
Being a clinician	−4.31	−13.57 to 4.95	0.34
Being a mentor	−6.5	−19.64 to 6.64	0.32
Experience in mentoring (years)	2.32	−5.63 to 10.26	0.55
Previous experience with student(s) who have had suicidal attempts	−1.47	−10.79 to 7.85	0.75
Previous exposure to suicide prevention program	−2.36	−23.05 to 18.33	0.82

^a^Total score change, defined as the total score of post-intervention minus pre-intervention.

## Discussion

This intervention study involved a sample of healthcare workers and lecturers in UKM to evaluate the effectiveness of an online gatekeeper suicide prevention training program among healthcare lecturers. Analysis of each individual item (53) in AdCARE-Q has helped us to have a better understanding of the outcome of this program which will allow us to have a targeted approach to improve suicide literacy in the future. The study outcome has shown that Online AdCARE effectively improves participants’ overall improvement in preventing suicide, especially in the Self-Efficacy (SE) domain. However, a lack of significant improvement following Online AdCARE in the domain of Declarative Knowledge (DK) was possibly due to ceiling effects; an already high baseline score within the domain among the participants.

The outcome for the SE domain was more encouraging. The baseline scores were relatively low as most participants agreed that they were hesitant to ask a person whether they are suicidal and were not confident in engaging with those who are suicidal. Lack of awareness of available resources in preventing suicide or steps to be taken in arranging appropriate help for those in need before the program is also a possible factor that may explain the relatively low baseline scores in this domain. This signifies the importance of having a suicide prevention gatekeeper training program among healthcare lecturers to effectively raise awareness and self-efficacy in dealing with suicidal cases due to their pivotal position in preventing suicide among healthcare students. Furthermore, this study has also shown that Online AdCARE is also beneficial for all healthcare workers even for those who were not mental health professionals as the program may empower those in improving their self-efficacy in suicide prevention by building their capacity as front liners to be able to identify and navigate help-seeking pathways. This program would be a beneficial continuing medical education (CME) topic in healthcare in the future (54) for its applicable lessons to their field of work (55), ease of technology (56), and short duration (57) that might be more feasible to be implemented in the real-world working environment. It is to reduce practical and logistic barriers such as time constraints within a high-pressure working environment in healthcare systems.

Our study findings are also supported by other studies where healthcare workers, even with reasonable literacy in suicide prevention, participants did not report feeling competent and confident enough in making suicide risk assessments (58–60). It is critical for participants to be able to provide appropriate clinical management of suicidal behavior other than being equipped with the knowledge of suicide prevention alone (61–63).

Incorporating role-plays, comprehensive feedback, and personalized suggestion is another factor in the significant improvement of attending Online AdCARE (64). This is consistent with another study, that has shown the added value of these hands-on experiences as the key factor that results in the significant improvement of the SE domain especially in endorsing a positive attitude, making a suicide risk assessment, developing treatment plans, and establishing rapport (65). A meta-analysis study on simulation learning such as role-play has also concluded that while an already skilled participant benefits from reflection phases during a simulation, a less skilled participant would benefit by learning through examples (66). As mentioned by Kolb (67), learning is a social experience and requires reflection. Furthermore, Online AdCARE is relevant to our participants in their scope of duty. With the increasing suicide trend especially among young adults, the need to acquire skills as gatekeepers in suicide prevention has facilitated their learning experience (68).

Further studies done locally has also shown benefit in simulation learning (69, 70) especially in learning a complex skill (71) such as engaging someone with suicidal thought. Trainers have reported a better understanding and recommend such a method as a tool to increase learners’ proficiency (72). Furthermore, this is in line with the new industrial revolution of Education 4.0 where simulation learning can empower learners to be competent, eventually leading to better patient safety (73).

Throughout the program, Online AdCARE adhered to the principle of safe messaging while facilitators intermittently checked on participants’ current emotional states for discussing suicide may be distressing to some individuals. The program also applied the standard of moderation in responsible suicide reporting by Duncan and Luce (53, 74). It suggests the practice of safe information by not including sensitive graphical details while practicing the proper use of tone and language when discussing suicide. Online AdCARE also included pathways in organizing help for a suicidal person within both general and local contexts following the standard of operating procedure in Malaysia, UKM, and HCTM. Finally, participants were also reminded of having postvention which is crucial to mitigate the negative effects of exposure to suicide (33, 44). All of these further improved participants’ SE in preventing suicide, especially for items in D3–D5 and B4.

One unanticipated finding is that the outcome of the program was not affected by any of the confounders where there were no significant differences even if the participants were clinicians or experienced mentors. This could mean that Online AdCARE would be beneficial in creating awareness and improving SE in suicide prevention for all healthcare workers regardless of their role and experience in supervising students. Excluded participants who were not from the study site showed interest in the program. They are healthcare workers who were interested in joining the program for its benefit in suicide prevention as they saw the recruitment poster on social media platforms. They were allowed to join the program but were not included in the study. By using the online method, Online AdCARE would also potentially lead to better outreach, feasibility, and lesser cost in providing an effective suicide prevention gatekeeper training program.

## Limitations

Despite the encouraging findings, this study is vulnerable to type II error due to the relatively small sample size. Our non-randomized sampling may lead to potential selection bias. There is also a lack of a control group that may provide a better understanding of the effectiveness of Online AdCARE. The self-rated questionnaire is also prone to cause bias from self-selection and may not reflect the true score of the participants. As mentioned in a previous study, health professionals tend to overestimate their self-assessment of competence (39). Furthermore, this study does not assess the long-term effectiveness of Online AdCARE due to the time constraint that we have in this study.

Our recommendation for future studies is that the study should utilize randomized sampling with a bigger sample size and a control group. An interviewer-rated questionnaire can also be developed to limit bias and better understand participants’ SE and DK. Other than that, it would also be beneficial to see the longitudinal effect of Online AdCARE on the participants after a few months of the intervention.

On top of that, we did not look into possible behavioral outcomes following Online AdCARE in this study. We recommend future studies look into these behavioral outcomes (29) objectively such as looking for a reduction in suicidal behavior in the community or an increase in referrals of suicide-related cases to hospitals.

## Conclusion

This study has shown that Online AdCARE is instrumental in improving self-efficacy of suicide prevention among its participants. This program has the potential to be expanded to a broader population of healthcare workers in low-and-middle income settings as it includes building capacity on hands-on skills as a gatekeeper while providing resources on postvention to participants. However, further investigations warrant a more rigorous study design, including a larger sample size, a control group, randomization, and a longer follow-up period to ascertain its effectiveness in the general healthcare population.

## Data availability statement

The original contributions presented in this study are included in the article/Supplementary material, further inquiries can be directed to the corresponding author.

## Ethics statement

The studies involving human participants were reviewed and approved by the Secretariat of Research and Innovation, Faculty of Medicine, National University of Malaysia. The patients/participants provided their written informed consent to participate in this study.

## Author contributions

LC and AR conceptualized and designed the study. LC, KP, PS, RM, SB, and HY supervised the data collection by AR. LC, AR, KP, and PS analyzed the data. AR drafted the manuscript. All authors reviewed and approved the final draft for submission.
