# Supplementary material for: Effectiveness of online advanced C.A.R.E suicide prevention gatekeeper training program among healthcare lecturers and workers in national university of Malaysia: A pilot study
Source: Front Psychiatry. 2023 Jan 19;14:1009754. doi: 10.3389/fpsyt.2023.1009754 (PMC9892857; doi:10.3389/fpsyt.2023.1009754)
Supplement: Supplementary file 1 [file Data_Sheet_1.PDF]

AdCARE-Q (Advanced C.A.R.E Suicide Prevention Gatekeeper Training Program Questionnaire) [Pre- and Post-program]

Advanced C.A.R.E Suicide Prevention Gatekeeper Training Program Questionnaire (AdCARE-Q) [Pre / Post]

**Section A**							
1	Date						
2	Profession						
3	Organization						
4	Email						
**Section B**							
How much do you know about the following subjects			Very Poor	Poor	Average	Good	Excellent
1	Knowledge on suicide prevention		1	2	3	4	5
2	Warning signs of suicide		1	2	3	4	5
3	Communicating with someone who is suicidal		1	2	3	4	5
4	How to arrange help for a suicidal person		1	2	3	4	5
**Section C**							
How far do you agree with the following statements:			Strongly Disagree	Disagree	Neutral	Agree	Strongly Agree
1	Depression is a potential suicide risk		1	2	3	4	5
2	People who are suicidal may not see a way out of their problems		1	2	3	4	5
3	A person who shows warning signs of suicide should be referred to a healthcare provider		1	2	3	4	5
4	Crisis helplines should be offered to a suicidal person		1	2	3	4	5
5	Farewell messages or asking for forgiveness unexpectedly are warning signs of suicide		1	2	3	4	5
6	People who express their suicidal ideation will not attempt suicide		1	2	3	4	5

Section D						
How far do you agree with the following statements:		Strongly Disagree	Disagree	Neutral	Agree	Strongly Agree
1	I have confidence in my abilities to recognize warning signs of suicide in people	1	2	3	4	5
2	I hesitate to ask a person whether they are suicidal	1	2	3	4	5
3	I have confidence in my abilities to arrange for help for someone who is suicidal	1	2	3	4	5
4	I am confident in discussing about safety planning with someone who is suicidal	1	2	3	4	5
5	I know where to seek resources for postvention services	1	2	3	4	5
